# The effect of pelvic pathology on uterine vein diameters

**DOI:** 10.1186/s13089-021-00212-y

**Published:** 2021-02-18

**Authors:** T. N. Amin, M. Wong, X. Foo, S.-L. Pointer, V. Goodhart, D. Jurkovic

**Affiliations:** Institute for Women’s Health, University College London Hospitals NHS Foundation Trust, 250 Euston Road, London, NW1 2PG UK

**Keywords:** Transvaginal ultrasound, Imaging, Uterine veins, Pathology, Adenomyosis, Fibroids, Pelvic venous congestion

## Abstract

**Background:**

Transvaginal ultrasound (TVS) is a sensitive tool for detecting various conditions that contribute to pelvic pain. TVS can be also used to assess blood flow and measure the size of pelvic veins. Pelvic venous congestion (PVC) is characterised by enlargement of the pelvic veins and has been recognised as a cause of chronic pelvic pain. The reference ranges for uterine venous diameter in women with normal pelvic organs have been established, but there is no information regarding the potential effect of pelvic pathology on the uterine venous diameters. The aim of this study was to examine the size of uterine venous plexus in women with evidence of pelvic abnormalities on TVS and to determine whether the reference ranges need to be adjusted in the presence of pelvic pathology.

A prospective, observational study was conducted in our gynaecological outpatient clinic. Morphological characteristics of all pelvic abnormalities detected on TVS and their sizes were recorded. The uterine veins were identified and their diameters were measured in all cases. The primary outcome measure was the uterine venous diameter. Regression analyses were performed to determine factors affecting the uterine venous size in women with pelvic pathology.

**Results:**

A total of 1500 women were included into the study, 1014 (67%) of whom were diagnosed with pelvic abnormalities. Women with pelvic pathology had significantly larger uterine venous diameters than women with normal pelvic organs (*p* < 0.01). Multivariable analysis showed that pre-menopausal status, high parity, presence of fibroids (*p* < 0.001) and Black ethnicity were all associated with significantly larger uterine vein diameters. Based on these findings modified reference ranges for uterine venous diameters have been designed which could be used for the diagnosis of PVC in women with uterine fibroids.

**Conclusions:**

Our findings show that of all pelvic pathology detected on TVS, only fibroids are significantly associated with uterine venous enlargement. Factors known to be associated with enlarged veins in women with normal pelvic organs, namely parity and menopausal status, also apply in patients with pelvic pathology. Future studies of uterine venous circulation should take into account the presence and size of uterine fibroids when assessing women for the signs of PVC.

## Background

Pelvic pain in women is an important health issue which accounts for a substantial proportion (20–40%) of all gynaecological outpatient referrals [1, 2]. Common gynaecological causes of pelvic pain include endometriosis, adhesions and chronic pelvic inflammatory disease (PID), as well as adenomyosis, fibroids and ovarian/adnexal cysts [3, 4]. Pelvic venous congestion (PVC), in which dilatation and stasis within the pelvic venous plexus occurs, has been postulated as a possible cause of pelvic pain [5, 6]. PVC is often used interchangeably with pelvic congestion syndrome, pelvic varicosities, pelvic venous insufficiency, and pelvic varicocele [3, 7]. Although the underlying pathophysiological mechanism is likely to be multifactorial, pelvic pain secondary to PVC is thought to result from direct stretching and activation of pain receptors in the vessel wall [8]. This concept remains controversial and causality has yet to be firmly established [7]. Furthermore, there are no universally accepted criteria as to what constitutes abnormally enlarged pelvic veins, and diagnostic characteristics vary across studies analysing this condition [1, 6, 9]. The seminal study by Beard et al., used venography to define moderately dilated pelvic veins as between 5 and 8 mm and severely dilated as > 8 mm [6]. Venography is an invasive technique which is only performed in specialist centres and it has been largely superseded by transvaginal ultrasound (TVS) as the primary method to assess the size of pelvic veins [10]. TVS is particularly suitable for assessing the uterine venous plexus, whilst the ovarian veins are much harder to examine due to their smaller size and variable position far from the transvaginal transducer [11]. The universal criteria for diagnosing PVC on TVS are lacking. Some authors define PVC based on ovarian venous diameter > 5 mm exclusively [6, 9]. Others apply the same cut-off to any pelvic vein [1, 12]. Additionally, features such as reflux within pelvic veins [10, 13, 14] irrespective of dilatation [15] have been used as diagnostic criteria of PVC. Given these discrepancies, defining PVC as a single entity and comparison between studies is challenging.

Pelvic pathology is thought to have a direct and indirect effect on vessel diameter. Fibroids and adenomyosis can cause enlargement of the uterus, which could be, in turn, associated with altered blood flow [16, 17]. Similarly, the presence of adnexal cysts may also increase blood supply to and from the pelvic organs, with a previous study demonstrating that women with PVC were more likely to have polycystic ovarian morphology (PCOM) on ultrasound^(18)^. Consequently, in women presenting with pelvic pain, dilated veins and additional pelvic pathology, determining whether the vessel dilation is a caused by that pathology, or is an independent contributor to the pain, is critically important. There is a paucity of data regarding the effects of pelvic pathology on the uterine venous system. The aim of this study was to assess the effect of uterine and ovarian/adnexal abnormalities on dimensions of the uterine veins in a large number of women attending for a gynaecological ultrasound examination.

## Methods

A prospective cross-sectional observational study was conducted in our general gynaecology clinic at a university teaching hospital in London, UK between August 2015 to December 2016. All women were seen by a single examiner (T.A.) who recruited them to the study and obtained their written consent. We excluded women who had previously undergone a hysterectomy, those who were unable to undergo TVS, women younger 18 years of age and those who declined to participate. We only included women who attended for their first visit. A full clinical history including demographic data, gynaecological, obstetric, medical and surgical history was recorded. Women were also asked about their current and past medical therapy, including the use of contraception. Women aged ≥ 45 who had been amenorrhoeic for at least 12 months and not using hormonal contraception were considered post-menopausal [19]. This also included women who had been diagnosed with early menopause and were using hormone replacement therapy (HRT).

All ultrasound examinations were performed by a level II single operator (T.A.) using a 4–9 MHz probe with a three-dimensional facility (Voluson E8, GE Medical Systems, Milwaukee, USA) in a standardised fashion, which has been previously described [20] and the findings of pelvic pathology were verified by a level III operator. Diagnoses of adenomyosis and fibroids were based on the previously established criteria [21]. The ovaries and adnexa were then examined and the presence of lesions were noted. Ovarian/adnexal pathology was defined as the presence of any type of ovarian/adnexal cysts, irrespective of their size or morphology except for simple cysts less than 2 cm in the mean diameter, which were considered functional in pre-menopausal and clinically insignificant in post-menopausal women. The morphology of the ovaries was documented as either normal, abnormal (presence of ovarian cyst) or PCOM. As part of the routine TVS examination, we examine all women for the presence of pelvic endometriosis. Endometriotic nodules were typically identified as stellate hypoechoic or isogenic solid lesions with irregular outer margins that were fixed to the surrounding structures and tender on palpation [22, 23]. Mobility of the pelvic organs was also assessed simultaneously by a combination of applying gentle pressure with the transvaginal probe and abdominal pressure with the examiner’s free hand. Once the pelvic organs had been examined, the uterine venous trunks were identified in the transverse plane originating at the level of the internal os. They were traced laterally in the base of the broad ligament up to the iliac vessels. As previously described [20], the largest trunk vessel was identified in the transverse plane and a straight segment of the vein was magnified. Callipers to measure the maximum antero-posterior uterine vein diameter, were placed between the inner walls of the vein 1–2 cm lateral to the uterus. The mean of three measurements was taken as the final value and the procedure was then repeated on the contralateral side.

### Statistical analysis

The sample size was based on showing a difference in uterine vein diameter between those with normal pelvic organs and those with pelvic pathology. As the focus of the research was on those with pelvic pathology, the design was such that there were twice as many women in this group. The uterine vein diameter values were estimated to have a standard deviation of 1.2 mm. A difference of 0.2 mm between the groups would be of clinical importance. We calculated that 425 women with normal pelvic organs and 850 women with pelvic pathology were required (1275 in total) to have power of 80% at alpha level 0.05. To allow for missing data in 15% of women, a total of 1500 women were recruited into the study. This calculation assumes only one uterine vein diameter measurement per person. However, as there are two measurements per woman, the actual power of the study to detect the size of effect indicated is > 80%.

Statistical analysis was performed using SPSS software to examine whether the presence and type of pelvic pathology affects the diameter of the uterine veins. As the distribution of the venous diameters followed a positively skewed distribution, the Wilcoxon matched-pairs test was used for comparing the left and right uterine venous diameters. Analyses were also performed to examine factors associated with the diameter size. For these analyses, the left and right diameters were combined together. Therefore, each woman contributed two measurements to the analysis. Due to the multiple measurements per women, the analysis was performed using multilevel linear regression. Two-level models were used with individual uterine veins nested within women. Due to the skewed distribution of the diameter values, the analysis was performed with the values on the log scale. The regression analysis was performed in two stages. Firstly, the separate association between each factor and diameter size was examined separately in a series of univariable analyses. Subsequently the joint association between the factors and outcome was examined in a multivariable analysis. A backwards selection procedure was used to retain only the statistically significant variables in the final model. Variables included in the model were: age, body mass index (BMI), ethnicity (categorical), parity (categorical: 0; 1; 2; 3; 4 +), menopausal status (categorical: pre-menopausal; post-menopausal), ultrasound diagnosis (categorical: normal; abnormal), presence of pathology (categorical: single; multiple); fibroid number (categorical: 0; 1–3; 4 +) and fibroid size (categorical: < 50 mm; > 50 mm). Lastly, in order to examine the predictors of the 95th centile, the analysis was performed using quantile regression. The factors found to be associated with diameter size from the multivariable analyses were accounted for in the final regression model.

### Ethical approval

The study was approved by the West Midlands-Solihull national ethics committee (14/WM/1266) and entered onto the ISRCTN registry (No. 48651822).

## Results

**Fig. 1:**
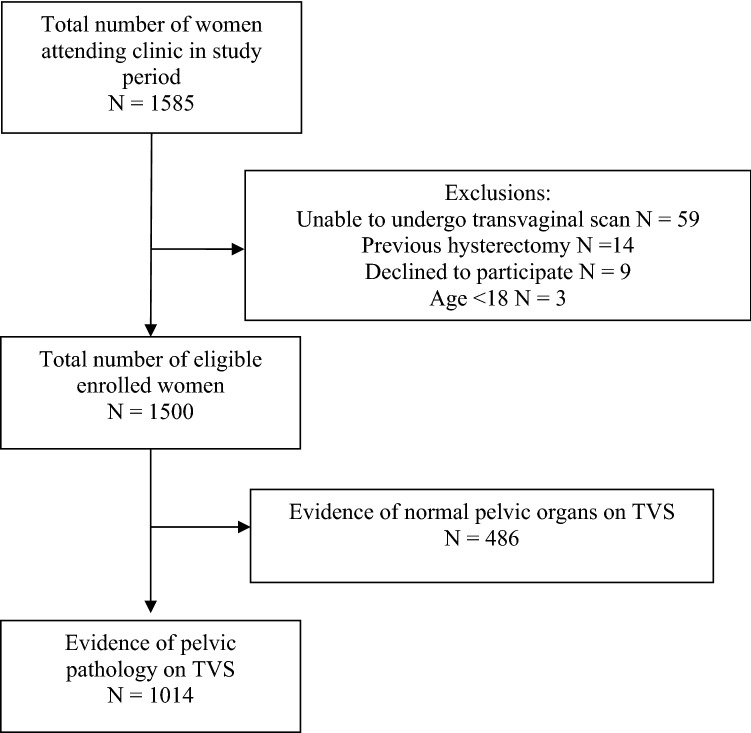
Flowchart of participants in the study

**Fig. 2:**
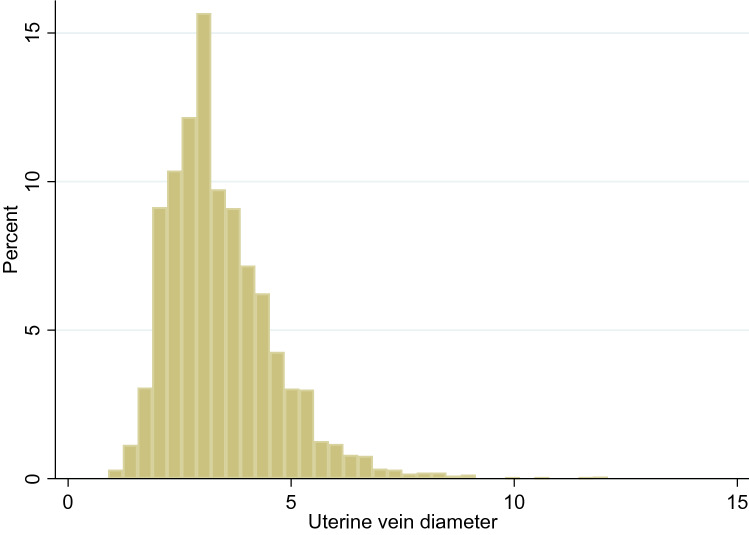
Distribution of uterine vein diameters (mm) in women with pelvic pathology on ultrasound

During the study period, 1500 women attended for clinical visits underwent TVS. 1014 (67.6%) had evidence of pelvic pathology on ultrasound examination (Fig. 1). Women’s demographic information is provided in Table 1, indication for TVS in Table 2 and the diagnoses following ultrasound assessment are shown in Table 3. The median diameters for the left and right uterine veins were 3.2 mm [IQR 2.6, 4.1] and 3.3 mm [2.6, 4.2], respectively. There was no significant difference in vein diameter between the left and right side (*p* = 0.07) and the combined values are presented in Fig. 2.

**Table 1:** Comparisons in demographic information between women with normal pelvic organs and those with pelvic pathology (*N* = 1500)

Variable	Category	Normal pelvic organs group (*N* = 486)	Pelvic pathology group (*N* = 1014)
Age	–	37.6 ± 8.5	43.8 ± 12.1
Parity	0	227 (47%)	451 (44.4%)
	1	95 (20%)	163 (16.1%)
	2	89 (18%)	195 (19.3%)
	3+	75 (15%)	205 (20.2%)
Menopausal status	Pre-menopausal	375 (77.1%)	777 (76.6%)
	Post-menopausal	111 (22.8%)	237 (23.4%)
BMI	–	22.3 (20.4, 25.3)	23.8 [21.3, 27.58]
Ethnicity	White	386 (79%)	656 (64.7%)
	Asian	34 (8%)	85 (8.4%)
	Black	25 (5%)	138 (13.6%)
	Middle Eastern	29 (6%)	72 (7.1%)
	Mixed/other	12 (2%)	63 (6.2%)

Summary statistics are: age ± standard deviation, median [inter-quartile range] or number (percentage)

**Table 2:** Indications for transvaginal ultrasound examination (*N* = 1500)

Indication for referral	*N* = 1500 N (%)
Irregular bleeding/periods	267 (21.2%)
Pelvic pain	209 (16.6%)
Heavy menstrual bleeding	185 (14.7%)
Post-menopausal bleeding	161 (12.8%)
Ovarian cysts/screening	131 (10.2%)
Infertility	68 (5.4%)
Fibroids	51 (4.1%)
Urogynaecological symptoms	46 (3.1%)
Vaginal/vulval symptoms	42 (2.8%)
Dysmenorrhoea	31 (2.5%)
Amenorrhoea/oligomenorrhoea	26 (2.1%)
Dyspareunia	24 (1.9%)
Irregular bleeding on HRT	20 (1.6%)
Other^a^	239 (15.9%)

^a^Small numbers of various indications < 10

**Table 3:** Ultrasound diagnoses for women presenting to the gynaecology clinic (*N* = 1500)

Variable	Category	Number N (%)
Ultrasound diagnosis	Normal pelvic organs	486 (32.4%)
	Pelvic pathology	1014 (67.6%)
	Single type of pathology	680 (67.1%)
	Multiple types of pathology	334 (32.9%)
Types of pathology	Adenomyosis	139 (13.7%)
	Fibroids	236 (23.2%)
	Ovarian/adnexal cysts	125 (12.3%)
	PCOM	130 (12.8%)
	Endometriosis	24 (2.4%)
	Other^a^	26 (2.6%)
	Combination of above	334 (32.9%)
Combination of ultrasound diagnoses	Adenomyosis and fibroids	139 (13.7%)
	Adenomyosis and ovarian/adnexal cysts	47 (4.6%)
	Adenomyosis and endometriosis	7 (0.7%)
	Adenomyosis and others^b^	14 (1.4%)
	Fibroids and ovarian cysts	44 (4.3%)
	Fibroids and endometriosis	7 (0.7%)
	Fibroids and others^b^	15 (1.5%)
	Adenomyosis, fibroids and endometriosis	15 (1.5%)
	Adenomyosis, fibroids and other^b^	31 (3.1%)
	Others^b^	15 (1.5%)

^a^Other denotes various diagnoses where *N* < 5

^b^Denotes various combinations with ovarian/adnexal cysts, PCOM, endometriosis or other pathologies if not already stated (*N* < 5)

**Fig. 3:**
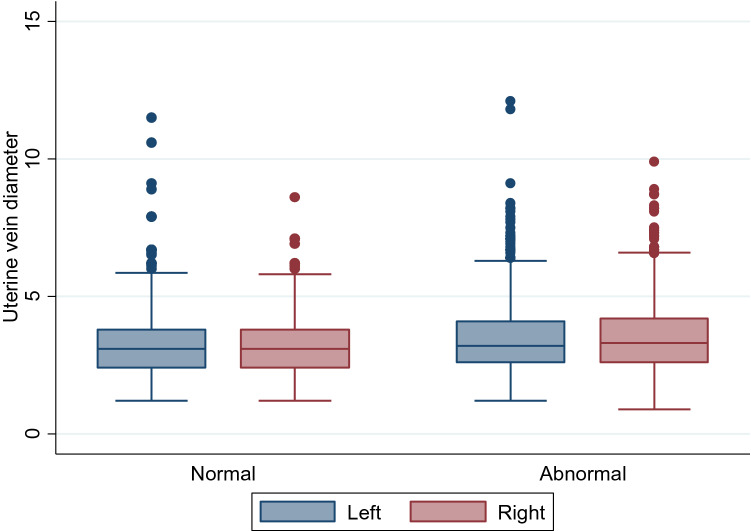
Differences in uterine vein diameter between all women with normal pelvic organs and those with pelvic pathology

Comparison between uterine diameters in women with normal pelvic organs on ultrasound and those with pelvic pathology is presented in Table 4. No significant differences between left and right uterine vein diameters were seen in any group. Venous dimeter was statistically larger in the group of patients with unselected pathology, although absolute difference in median values was small (0.1–0.2 mm) (Fig. 3). A statistically significant difference was maintained when only pre-menopausal women were included, but no difference in uterine venous diameter was observed in post-menopausal women with and without pelvic pathology.

**Table 4:** Comparisons of uterine vein diameters between women with normal pelvic organs and with pelvic pathology (*N* = 1500)

**Group**	**Uterine vein**	**Normal pelvic organs**		**Pelvic pathology**		***P value***
		**N**	**Median (IQR)**	**N**	**Median (IQR)**	
All	Left	486	3.1 (2.4, 3.8)	1014	3.2 (2.6, 4.1)	*0.009*
	Right	486	3.1 (2.4, 3.8)	1014	3.3 (2.6, 4.2)	* < 0.001*
Pre-menopausal	Left	375	3.1 (2.5, 3.8)	777	3.3 (2.7, 4.2)	*0.02*
	Right	375	3.1 (2.5, 3.8)	777	3.3 (2.7, 4.2)	* < 0.001*
Post-menopausal	Left	111	2.8 (2.2, 3.8)	237	3.1 (2.5, 4.0)	0.28
	Right	111	2.9 (2.2, 3.5)	237	3.0 (2.4, 3.8)	0.13

Table 5 shows the variables examined by univariable analyses. Age, parity, pre-menopausal status, non-White ethnicity, the presence adenomyosis, fibroids and combined pathology were associated with significantly larger venous diameters on univariate analysis. Body mass index, the presence of PCOM, ovarian/adnexal cysts and endometriosis were not statistically significant.

**Table 5:** Univariable analysis of factors affecting uterine venous diameter (*N* = 1014)

Variable		Category /term		Diameter Median [IQR]	Ratio (95% CI)		*P* value	
Age ^(*)^		Linear term		-	1.84 (1.39, 2.44)		* < 0.001*	
		Squared term			0.89 (0.84, 0.95)			
		Cubic term			1.01 (1.00, 1.01)			
BMI ^(*)^		Linear term		-	1.00 (0.98, 1.02)		0.91	
Ethnicity		White		3.2 [2.6, 4.1]	1		* < 0.001*	
		Asian		3.2 [2.7, 3.8]	0.99 (0.93, 1.06)			
		Black		3.6 [2.9, 4.5]	1.12 (1.06, 1.18)			
		Middle Eastern		3.4 [2.7, 4.4]	1.05 (0.98, 1.13)			
		Mixed/other		3.0 [2.6, 4.0]	0.96 (0.89, 1.03)			
Parity		0		3.0 [2.4, 3.7]	1		* < 0.001*	
		1		3.3 [2.6, 4.0]	1.09 (1.04, 1.13)			
		2		3.4 [2.7, 4.4]	1.15 (1.10, 1.19)			
		3		3.6 [2.8, 4.7]	1.20 (1.14, 1.26)			
		4 +		3.7 [3.0, 4.7]	1.24 (1.17, 1.32)			
Menopausal		Pre		3.2 [2.6, 4.1]	1		* < 0.001*	
Status		Post		3.0 [2.3, 3.8]	0.91 (0.88, 0.94)			
Diagnosis		Normal		3.1 [2.4, 3.8]	1		* < 0.001*	
		Abnormal		3.3 [2.6, 4.1]	1.07 (1.03, 1.10)			
Pathology		Single		3.2 [2.5, 4.0]	1		*0.002*	
		Multiple		3.3 [2.7, 4.2]	1.06 (1.02, 1.09)			
Adenomyosis		No		3.1 [2.5, 3.9]	1		* < 0.001*	
		Yes		3.4 [2.7, 4.3]	1.08 (1.05, 1.12)			
Fibroids		No		3.1 [2.5, 3.9]	1		* < 0.001*	
		Yes		3.4 [2.7, 4.2]	1.08 (1.04, 1.11)			
Fibroids		No fibroids		3.1 [2.5, 3.9]	1		*0.01*	
(by number)		1–3		3.3 [2.7, 4.3]	1.07 (1.04, 1.11)			
		4 +		3.4 [2.7, 4.2]	1.09 (1.03, 1.15)			
Fibroids		No fibroids		3.1 [2.5, 3.9]	1		* < 0.001*	
(by mean		≤ 50 mm		3.3 [2.7, 4.1]	1.05 (1.02, 1.09)			
diameter)		> 50 mm		3.9 [3.1, 4.7]	1.23 (1.15, 1.31)			
Fibroid		Linear term		-	0.99 (0.96, 1.02)		* < 0.001*	
Diameter ^(+) (*)^		Squared term			1.004 (1.001, 1.007)			
Ovarian/adnexal cyst		No		3.2 [2.6, 4.0]	1		0.45	
		Yes		3.2 [2.6, 4.1]	1.02 (0.98, 1.06)			
PCOM		No		3.2 [2.6, 4.0]	1		0.06	
Endometriosis		Yes		3.1 [2.5, 3.9]	0.96 (0.91, 1.00)		0.35	
		No		3.2 [2.6, 4.0]	1			
		Yes		3.3 [2.7, 4.2]	1.03 (0.97, 1.09)			

(*) Ratios given for a 10-unit increase in variable

(+) Analysis for subgroup of 488 patients with fibroids only

**Fig. 4:**
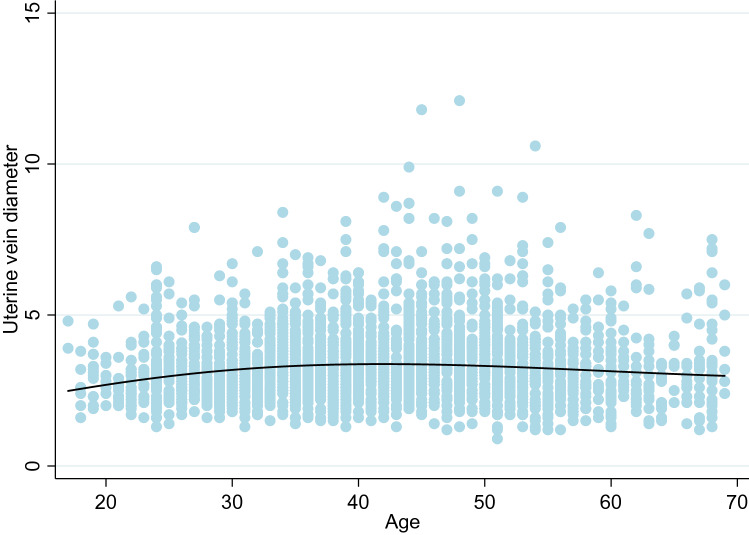
The effect of age on uterine vein diameter (mm)

The results for age followed a non-linear relationship, with venous diameter increasing with age up to around 40 years, after which there was a decrease in size (Fig. 4). This finding suggests the effect of age may be a confounding factor for menopausal status.

**Fig. 5:**
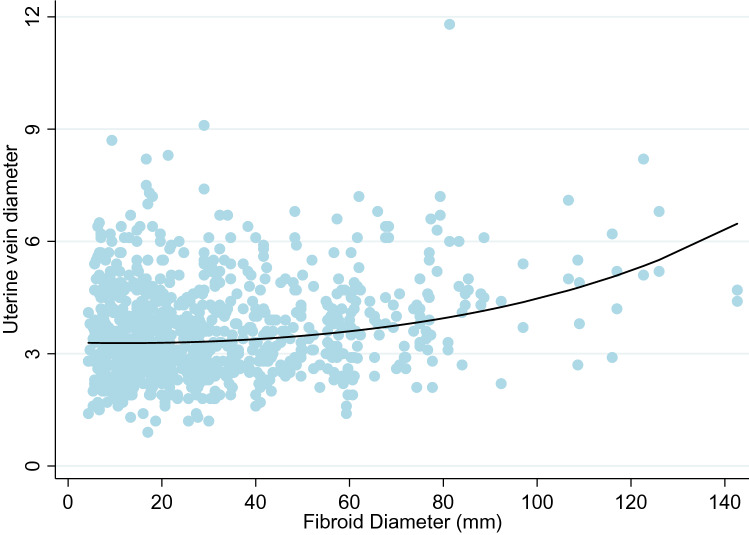
Relationship between increasing the mean fibroid dimeter (mm) and uterine vein diameter (mm)

Women with a parity of four or higher had venous diameter sizes that were, on average, 24% higher than women with a parity of zero. The occurrence of both adenomyosis and fibroids were associated with an 8% increase in venous diameters. Fibroids were also examined by number and the mean diameter of the largest fibroid. Number of fibroids was not a statistically significant variable; however, size of the fibroid was (Fig. 5).

Table 6 summarises the results of the multivariable analyses, in which two sets of analyses were performed. Model 1 reports with fibroids as a binary measure, and Model 2 uses fibroids split by size. Both sets of multivariable analyses suggested that parity, pre-menopausal status, Black ethnicity and fibroids were statistically significant, and thus could be considered independently associated with larger venous diameters. After adjusting for these variables, there was no longer any significant association between age, multiple pathology or adenomyosis all of which were found to be significant in the univariable analyses. As in the univariable analyses, women of higher parity had larger veins, and post-menopausal women again had smaller diameters than pre-menopausal women.

**Table 6:** Multivariable associations affecting uterine venous diameter (*N* = 1014)

Model	Variable	Category	Ratio (95% CI)	*P* value
1	Parity	0	1	< 0.001
		1	1.12 (1.06, 1.18)	
		2	1.17 (1.12, 1.23)	
		3	1.26 (1.19, 1.33)	
		4 +	1.29 (1.20, 1.38)	
	Menopausal status	Pre	1	< 0.001
		Post	0.84 (0.80, 0.88)	
	Ethnicity	White	1	0.02
		Asian	0.95 (0.89, 1.01)	
		Black	1.06 (1.01, 1.12)	
		Middle Eastern	0.98 (0.91, 1.05)	
		Mixed/other	0.95 (0.88, 1.02)	
	Fibroids	No	1	0.003
		Yes	1.06 (1.02, 1.09)	
2	Parity	0	1	< 0.001
		1	1.13 (1.07, 1.19)	
		2	1.18 (1.13, 1.24)	
		3	1.27 (1.20, 1.34)	
		4 +	1.31 (1.22, 1.40)	
	Menopausal status	Pre	1	< 0.001
		Post	0.85 (0.81, 0.88)	
	Ethnicity	White	1	0.03
		Asian	0.94 (0.88, 1.00)	
		Black	1.04 (0.99, 1.10)	
		Middle Eastern	0.98 (0.91, 1.05)	
		Mixed/other	0.94 (0.87, 1.01)	
	Fibroids	No fibroids	1	< 0.001
	(by mean	≤ 50 mm	1.03 (0.99, 1.07)	
	diameter)	> 50 mm	1.22 (1.14, 1.30)	

When fibroids were considered as a binary measure those with fibroids had 6% larger venous diameters than those with no fibroids. When divided by the size of the largest fibroid, those with the largest fibroids (mean diameter > 50 mm) had on average 22% larger venous diameters compared to those with no fibroids. There was relatively little difference in venous size between those with smaller fibroids and those with no fibroids. Based on the multivariable results, the 50th and 95th venous diameter size can be predicted by the following equations:

$$\begin{aligned} {5}0{\text{th centile }} =\, & {3}.0 \, {-} \, 0.{5 }\left( {{\text{if}} {\text{post}} - {\text{menopausal}}} \right) \, + \, 0.{5 }\left( {\text{if parity 1}} \right) \, \\ &+ \, 0.{6 }\left( {\text{if parity 2}} \right) \, + { 1}.0 \, \left( {\text{if parity 3}} \right) \, \\ &+ \, 0.{9 }\left( {\text{if parity 4}} \right) \, + \, 0.{1 }\left( {{\text{if fibroids}} \le { 5}0} \right) \\ & + \, 0.{7} \left( {{\text{if fibroids }} > { 5}0} \right) \, {-} \, 0.{3 }\left( {\text{if Asian ethnicity}} \right) \,\\ & + \, 0.{3 }\left( {\text{if Black ethnicity}} \right) \,\\ & {-} \, 0.{1 }\left( {\text{if Middle Eastern ethnicity}} \right)\\ & - \, 0.{3 }\left( {{\text{if mixed}}/{\text{other ethnicity}}} \right), \\ \end{aligned}$$

$$\begin{aligned} {\text{95th centile }} =\, & {5}.0 \, {-} \, 0.{6} \left( {\text{if post - menopausal}} \right) \\ &+ \, 0.{9} \left( {{\text{if parity}} {1}} \right) \, + { 1}.0 \left( {{\text{if parity}} {2}} \right) \\ &+ { 1}.{6} \left( {{\text{if parity}} {3}} \right) + { 2}.{8} \left( {{\text{if parity}} {4}} \right)\\ & + \, 0.{1} \left( {{\text{if fibroids}} \le {5}0} \right)\\ & + { 1}.{3} \left( {{\text{if fibroids}} > {5}0} \right) \,\\ & - 0.{9 }\left( {\text{if Asian ethnicity}} \right) \, \\ &+ \, 0.0 \, \left( {\text{if Black ethnicity}} \right) \,\\ & {-} \, 0.{2 }\left( {\text{if Middle Eastern ethnicity}} \right) \, \\ &- 0.{9 }\left( {\text{if mixed/other ethnicity}} \right). \\ \end{aligned}$$

An example of how these equations could be used in clinical practice is given below:

The 50th and 95th centile measurements for a pre-menopausal woman of Asian ethnicity, with a parity of 4 and fibroids > 50 mm would be:

$$\begin{aligned} {5}0{\text{th centile }} =& { 3}.0 \, + \, 0.{9 }\left( {\text{parity 4}} \right) \, \\ &+ \, 0.{7 }\left( {{\text{fibroids }} > { 5}0\,{\text{mm}}} \right) \, \\ &- \, 0.{3 }\left( {\text{Asian ethnicity}} \right) \, = { 4}.{3}\,{\text{mm,}} \end{aligned}$$

$$\begin{aligned} {\text{95th centile }} =& { 5}.0 \, + { 2}.{8 }\left( {\text{parity 4}} \right) \, + { 1}.{3 }\left( {{\text{fibroids }} > { 5}0{\text{mm}}} \right) \,\\ & {-} \, 0.{9 }\left( {\text{Asian ethnicity}} \right) \, = { 8}.{\text{2mm}}{.} \end{aligned}$$

The 50th and 95th centile measurements for a post-menopausal woman of White ethnicity, with a parity of 2 and fibroids ≤ 50 mm would be:

$$\begin{aligned} {5}0{\text{th centile }} =& { 3}.0 \, {-} \, 0.{5 }\left( {{\text{post}} - {\text{menopausal status}}} \right) \, \\ &+ \, 0.{6 }\left( {\text{parity 2}} \right) \, + \, 0.{1 }({\text{fibroids}} \le {5}0{\text{mm}}) \, \\ &+ \, 0.0 \, \left( {\text{White ethnicity}} \right) \, = { 3}.{\text{2mm,}} \end{aligned}$$

$$\begin{aligned} {\text{95th centile }} =& { 5}.0 \, {-} \, 0.{6 }\left( {{\text{post}} - {\text{menopausal status}}} \right) \,\\ & + { 1 }\left( {\text{parity 2}} \right) \, + \, 0.{1}({\text{fibroids}} \le {5}0{\text{mm}}) \, \\ &+ \, 0.0 \, \left( {\text{White ethnicity}} \right) \, = { 5}.{\text{5mm}}{.} \end{aligned}$$

## Discussion

This study has shown that pre-menopausal status, high parity, Black ethnicity and the presence of fibroids are all associated with larger uterine venous diameters on ultrasound examination. The number of fibroids was less important than the size with fibroid measuring > 50 mm in the mean dimeter having particularly strong effect on pelvic venous circulation.

In our recent study we described a reference range for uterine vein diameters in women with normal pelvic organs [20]. We found that vessel diameter was dependent on parity and menopausal status, with multiparous and pre-menopausal women having larger veins. The current study confirmed that these factors have a significant effect on the uterine vein diameters in women with pelvic pathology as well. These findings are in agreement with those applied by Beard et al. [6] who identified pre-menopausal status and multiparity as important characteristics in the diagnosis of PVC.

A new finding from this study is that fibroids are associated with larger uterine venous diameters. This differs from the study by Beard et al. [6] who found no association between pelvic pathology and venous diameter; however, their study excluded women with fibroids.

Pelvic pathology, such as large fibroids have been shown to increase perfusion to the uterus [17]. This, in turn, has to result in concomitant increase in venous drainage as well. Fibroids were the most commonly detected pathology in our population which is similar to the prevalence outlined by Cramer and Patel [24]. Fibroids are also more common in Black women [25]. In our multivariable analysis, women of Black ethnicity were found to have 4–6% larger uterine venous diameters compared to other ethnic groups. It is possible that this could be a confounder, however both ethnicity and fibroids were adjusted for in the final regression model.

The presence of endometriosis and PCOM were comparable with findings reported by Naftalin et al., which was conducted in the same unit, several years before [26]. Our prevalence of adenomyosis at 38.3% was higher than the previously reported figure of 20% [26], which may be secondary to improved diagnostic accuracy due to better quality ultrasound equipment and better awareness of the diagnostic criteria [21]. Overall, the mix and frequency of pelvic pathology recorded in our study mirrors that of previously published studies, suggesting our results are likely to be widely applicable.

Previous studies have shown an association between PCOM and PVC [18]. We could not confirm that and in our study women diagnosed with PCOM and adnexal cysts did not have statistically larger pelvic vessels.

Focusing solely on the uterine vein allowed us to carry out highly reproducible measurements which improved quality of our data. The uterine vein lies in close proximity to the TVS probe and can be traced in its entirety from its origin at the level of the internal os to the iliac veins. Others have reported diameters of the ovarian, iliac and para-uterine veins, which differed depending when measured using different diagnostic technique such as venography, TVS or CT [1, 6, 9, 10, 12, 27, 28]. The ovarian vein originates within the broad ligament and drains into the inferior vena cava on the right and renal vein on the left. Whilst ovarian vein imaging by venography or CT may be reproducible, there are technical issues with imaging ovarian veins on TVS and transabdominal ultrasound as mentioned previously [11]. One of the limitations of measuring only the uterine vein is that we have not addressed the overall pelvic vascular environment by not commenting on the presence of collateral networks. This can be can be incorporated into future studies.

As the venous circulation is dynamic, various factors such as hydration and cardiac output can affect venous filling ^(29)^. It is not possible to fully account for these variables and, although they could affect the vessel diameters, we do not think this should have a negative effect on the validity of our findings. We did not control for the phase of the menstrual cycle, but this has previously been shown not to have a significant effect the pelvic venous circulation [6, 20].

We have previously published the reference ranges for uterine venous diameters which were adjusted for women’s menopausal status and parity. The results of this study showed that further adjustments are needed when uterine fibroids > 50 mm in average diameter are present. In view of that, we provide a mathematical formula which could be used to define 50th and 95th centile for uterine venous diameters in women with pelvic pathology, taking into account the effect uterine fibroids have on the vasculature. This is important as using the reference ranges developed in women with normal pelvic organs would lead to over-diagnosis of PVC in women with uterine fibroids. For example, according to our data, the upper value for the uterine vein diameter in pre-menopausal multiparous women with fibroids > 50 mm is 4.9 mm. This would be classified as being abnormally large using the criteria set out by Beard et al. A woman with the same demographics at the 95th centile would have an expected uterine venous diameter of 9 mm which would be considered severely abnormal by most of the previously published diagnostic criteria.

Our study was carried out in a single centre and all examinations were performed by a single operator. In view of that it is important to confirm that our findings could be reproduced in other centres by operators of different levels of experience. In addition, further studies are needed to explore possible association of PVC and chronic pelvic pain using our adjusted cut-off levels. The validity of our findings could be checked by examining women prior and following myomectomy for large fibroids. This would help to establish whether the increased diameter of pelvic veins is indeed due to the increased blood supply needed to perfuse large fibroids or whether it could be only coincidental to their presence.

## Conclusion

Women with pelvic pathology on ultrasound have in general larger uterine veins than women with normal pelvic organs, with fibroids having the greatest effect of their size. This highlights the need to take uterine fibroids into consideration when assessing the pelvic venous circulation. Our findings show that no single value can be used to diagnose dilated pelvic veins in women with evidence of uterine fibroids. We therefore provide a formula to help clinicians and researchers to define the 50th and 95^th^ centile for venous diameters which takes into account all factors with significant effect on uterine venous diameters. The use of our formula would facilitate evidence-based ultrasound diagnosis of enlarged pelvic veins and may facilitate future research into significance of PVC in women presenting with chronic pelvic pain.

## Key message

Pre-menopausal status, higher parity and uterine fibroids are all associated with increased diameters of pelvic veins. These factors need to be taken into consideration when diagnosing pelvic venous congestion.

## Data Availability

The datasets generated and/or analysed during the current study are not publicly available due to ongoing studies, but are available from the corresponding author on reasonable request.

## Abbreviations

BMI: Body mass index
HRT: Hormone replacement therapy
PID: Pelvic inflammatory disease
PCOM: Polycystic ovarian morphology
PVC: Pelvic venous congestion
TVS: Transvaginal ultrasound scan
